# Unmasking the Silent Glomus Tumor of the Index Finger—A Case Report and Literature Review

**DOI:** 10.1002/ccr3.71849

**Published:** 2026-01-12

**Authors:** Eesha Iqbal, Muhammad Hamza, Tariq Rashid, Laiba Nasir, Fareena Ambreen, Fazeela Bibi, Khalil El Abdi, Said Hamid Sadat

**Affiliations:** ^1^ Department of Dermatology FMH College of Medicine and Dentistry Lahore Pakistan; ^2^ Department of Medicine Saidu Medical College Swat Pakistan; ^3^ Department of General Surgery FMH College of Medicine and Dentistry Lahore Pakistan; ^4^ Combined Military Hospital Lahore Pakistan; ^5^ Department of Medicine Khyber Girls Medical College Peshawar Pakistan; ^6^ Jinnah Medical and Dental College Karachi Pakistan; ^7^ Faculty of Medicine and Pharmacy of Rabat Mohammed V University Rabat Morocco; ^8^ Kabul University of Medical Sciences Abu Ali Ibn Sina Kabul Afghanistan

**Keywords:** benign tumor, case report, finger pain, glomus tumor, hand surgery, subungual

## Abstract

Glomus tumors are rare, benign neoplasms of the finger, notorious for causing debilitating pain and significant diagnostic delays. We present the case of a 62‐year‐old female with a 15‐year history of chronic, severe left index finger pain that was exacerbated by cold and pressure. Unusually, her pain radiated proximally to the shoulder, which contributed to a prolonged misdiagnosis. Despite a previously inconclusive MRI, a clinical diagnosis was made based on pathognomonic historical features. Surgical excision of a 1‐mm subungual tumor provided immediate and complete symptomatic relief, with histopathology confirming the diagnosis of a glomus tumor. This case highlights the diagnostic challenges posed by glomus tumors, especially with atypical presentations such as referred pain. It underscores the primacy of clinical acumen over imaging in certain contexts and reinforces that surgical excision is curative, emphasizing the need for heightened clinical suspicion to prevent years of patient suffering.

## Key Clinical Message

Subungual glomus tumors are rare causes of chronic, debilitating finger pain, frequently misdiagnosed for 7–15 years. Timely recognition, even with atypical radiation, is crucial. Surgical excision offers patients immediate and complete pain relief.

## 1 Introduction

Glomus tumors are benign neoplasms that arise from neuromyoarterial glomus bodies, which are highly concentrated in the digits and are involved in thermoregulation. Although they account for only 1%–5% of all hand tumors, their presentation with a classic triad of severe paroxysmal pain, exquisite point tenderness, and cold sensitivity can cause significant patient distress. The majority are solitary lesions found in the subungual region of the finger [1, 2].

Despite these characteristic features, glomus tumors are frequently misdiagnosed, leading to years of unnecessary suffering [3]. While these tumors are well‐known for causing diagnostic delays, often averaging 7–15 years, presentations with atypical features such as proximally radiating pain can further complicate recognition [4]. Here, we present a case of a subungual glomus tumor with a 15‐year diagnostic delay and an uncommon pattern of pain radiation to the shoulder, highlighting the clinical pearls and pitfalls in its diagnosis and management.

## 2 Case History

A 62‐year‐old right‐hand dominant female with a history of type 2 diabetes mellitus presented with a 15‐year history of progressively worsening pain in her left index finger. The symptoms began with mild, intermittent pain triggered by touch and cold. Over the years, the pain has evolved to become severe, sharp, and spontaneous, often waking her from sleep. For approximately the last 5 years, the pain has begun radiating proximally to her left shoulder. She had consulted multiple healthcare providers over the 15‐year period without receiving a definitive diagnosis. An MRI performed 3 years prior was reported as inconclusive.

**FIGURE 1:**
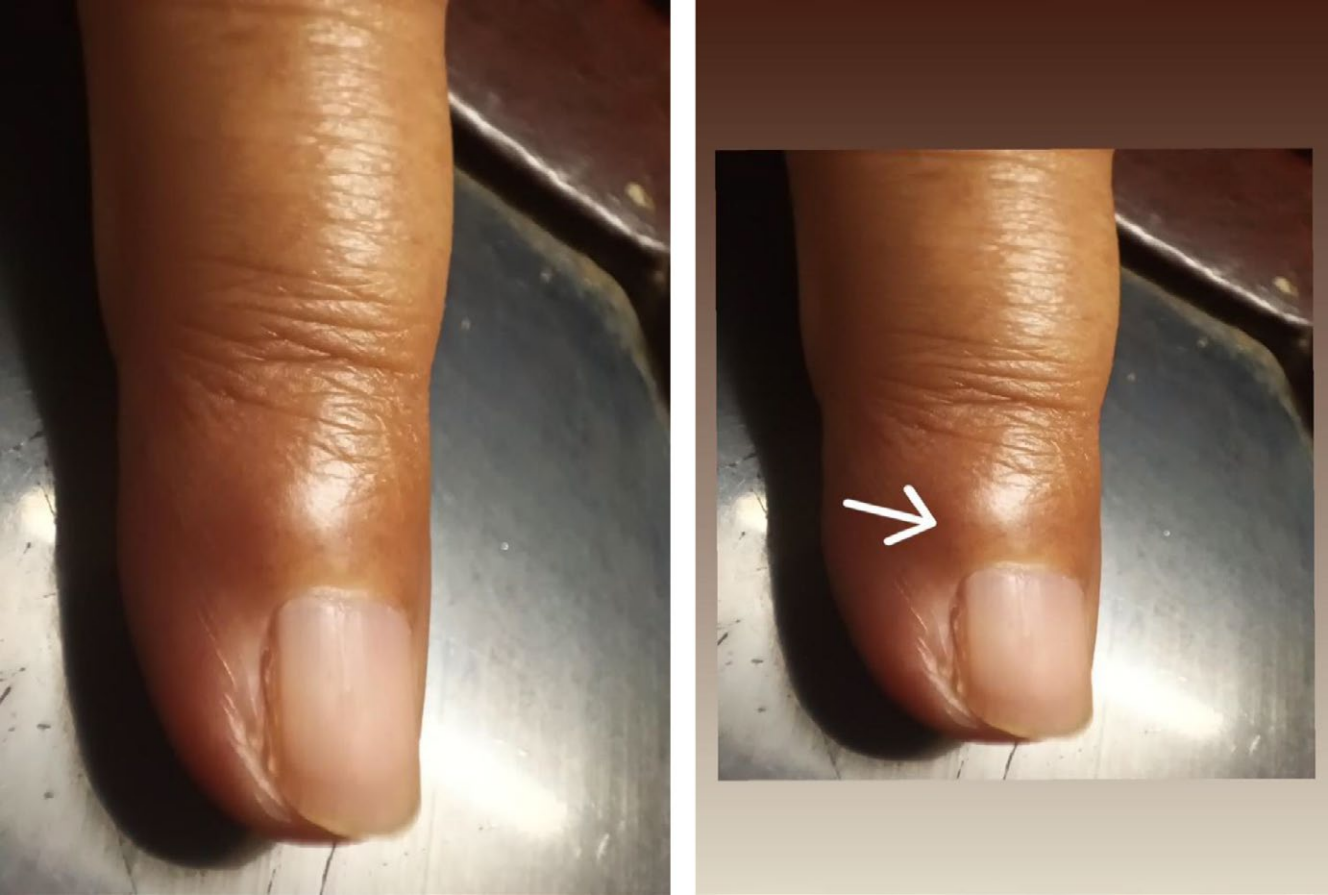
Clinical photograph of the patient's left index finger at presentation. The digit demonstrates a notable absence of overt pathological signs, such as discoloration or nail plate deformity, which contributed to the diagnostic delay. The arrow precisely indicates the subungual region of maximum pinpoint tenderness, corresponding to a subtle, palpable nodularity consistent with the underlying tumor.

On physical examination, the left index finger and nail appeared normal upon gross inspection, with no discoloration or deformity. Palpation of the medial aspect of the subungual area revealed a subtle, firm nodularity (Figure 1). Applying gentle pressure with a blunt probe over this specific point elicited excruciating pain, consistent with a positive Love's pin sign. This tenderness was highly localized, as palpation of the surrounding finger pulp and adjacent areas did not cause pain.

## 3 Diagnostic Assessment and Differential Diagnosis

Although formal provocative maneuvers such as Hildreth's test were not documented in prior evaluations, the patient's history was pathognomonic, including excruciating pinpoint tenderness (a positive Love's pin sign equivalent), paroxysmal pain, and severe cold sensitivity. Laboratory investigations were not performed, as hematological and inflammatory markers are typically normal in patients with glomus tumors and were not expected to contribute to the diagnosis.

An MRI of the left hand, performed at an outside institution 3 years prior to presentation, was reported as inconclusive. Technical details such as field strength and sequence parameters were not available for review. Given the tumor's ultimate size of approximately 1 mm, it is likely that the lesion was below the detection threshold of a standard, non‐dedicated imaging protocol. A plain radiograph of the finger was also unremarkable, showing no osseous erosion.

The differential diagnosis for chronic, localized fingertip pain is broad. Neuromas or complex regional pain syndrome (CRPS) were considered but were less likely given the well‐circumscribed, pinpoint nature of the tenderness and the absence of autonomic signs. Gouty arthritis was unlikely due to the lack of inflammatory joint signs and normal joint appearance. Other benign tumors, such as subungual exostosis or osteoid osteoma, were excluded by the normal radiograph. Vascular malformations or hemangiomas could present similarly but were not supported by the clinical examination. Finally, amelanotic subungual melanoma can present as a painful papule, but the 15‐year history of stable, albeit worsening, symptoms made a malignant process less probable.

Based on the classic history and pinpoint tenderness, a provisional diagnosis of a glomus tumor was made, and the patient was referred for surgical excision.

## 4 Therapeutic Intervention

Given the definitive clinical signs despite inconclusive imaging, surgical excision was planned. The procedure was performed under a digital nerve block with tourniquet control for hemostasis. A transungual approach was utilized, wherein a vertical incision was made, and the nail plate was carefully elevated to expose the underlying nail bed. This approach provides excellent visualization of the tumor.

A small, encapsulated, reddish‐blue nodule measuring approximately 0.1 × 0.1 cm was identified directly beneath the nail root. Meticulous excision of the tumor was performed, with the use of surgical loupe magnification to ensure clear margins and complete removal. The tumor bed was then curetted to minimize the risk of recurrence, which is primarily associated with incomplete excision. The nail plate was repositioned, and the wound was closed with fine sutures.

**FIGURE 2:**
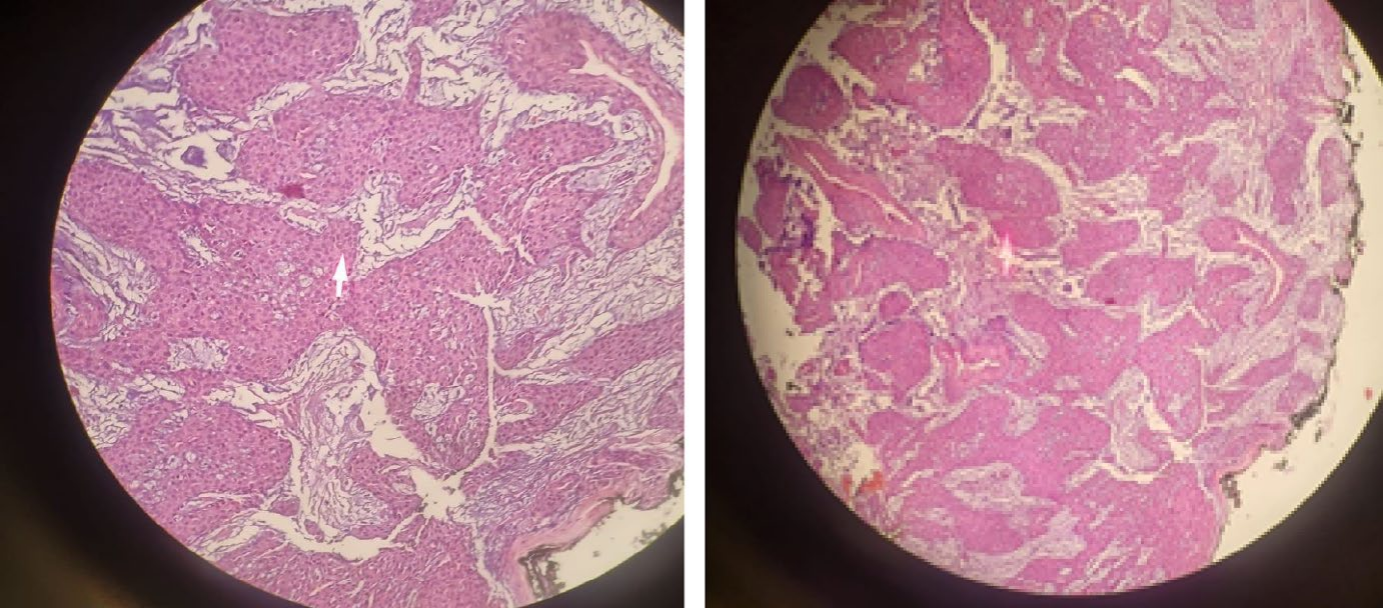
Histopathological confirmation of a benign glomus tumor. This photomicrograph reveals the characteristic architecture of the lesion, composed of solid sheets of uniform, round glomus cells with bland, centrally located oval nuclei and moderate eosinophilic cytoplasm. These cells are arranged around small vascular channels. Crucially, there is no evidence of nuclear atypia, mitotic activity, or necrosis, confirming the benign nature of the neoplasm (Hematoxylin and Eosin stain; 10X magnification).

The excised specimen was sent for histopathological analysis. Microscopic examination revealed well‐circumscribed, solid sheets of uniform round cells with bland, oval nuclei and moderate eosinophilic cytoplasm, surrounding vascular channels (Figure 2). There was no evidence of mitotic activity, nuclear atypia, or necrosis. These findings confirm the diagnosis of a benign glomus tumor.

## 5 Outcome and Follow‐Up

The patient experienced immediate and complete resolution of her severe, long‐standing symptoms, including both the localized finger pain and the referred shoulder pain, in the immediate postoperative period. At the first postoperative visit, 10 days after surgery, the surgical site was clean, dry, and healing appropriately, and the sutures were removed.

At her final follow‐up, 6 months postoperatively, she remained entirely asymptomatic with no recurrence of pain, tenderness, or cold sensitivity. There was no clinical evidence of local tumor recurrence, and the nail plate had regrown without significant deformity or discoloration, which can be a complication of the transungual approach. This outcome aligns with literature reports where complete surgical excision is curative and provides lasting relief.

## 6 Discussion

This case of a subungual glomus tumor exemplifies two of the most significant challenges in its management: the potential for profound diagnostic delay and the confounding nature of an atypical pain presentation.

Glomus tumors, composed of modified smooth muscle cells from the neuromyoarterial glomus body, are most prevalent in the fingertips, where these structures are highly concentrated. While their clinical features can be pathognomonic, their overall rarity frequently leads to misdiagnosis, causing years of patient suffering [5, 6, 7].

The 15‐year delay in diagnosis experienced by our patient, while extensive, aligns with the upper limit of the 7‐to‐15‐year range commonly cited in the literature [4]. Such prolonged delays are often attributed to the tumor's small size and the nonspecific nature of early symptoms, which can be mistaken for arthritis, neuromas, or CRPS [8]. Indeed, one report documents a diagnostic delay of up to 40 years, underscoring the severity of this clinical problem [3]. A more unusual feature of this case was the radiation of pain to the ipsilateral shoulder, a symptom that is rarely documented. The classic presentation involves sharply localized digital pain, and while proximal radiation up the arm is sometimes mentioned, specific referral to the shoulder is uncommon. This atypical pain pattern may have further contributed to the diagnostic inertia by diverting clinical suspicion toward cervical spine or primary shoulder pathology.

This case also powerfully reinforces the primacy of clinical acumen over imaging in the diagnosis of glomus tumors. The patient's MRI, performed 3 years prior, was reported as inconclusive. Although MRI can demonstrate very high sensitivity in detecting these lesions, its efficacy is highly dependent on technique and tumor size. Imaging studies are known to yield false‐negative results, particularly for tumors smaller than 3 mm in diameter [5, 9, 10, 11, 12]. The lesion in this case, measuring only 1 mm, almost certainly fell below the detection threshold of a standard, non‐dedicated imaging protocol, explaining the negative finding. This underscores a crucial teaching point: a compelling clinical history characterized by the classic triad of paroxysmal pain, pinpoint tenderness, and cold intolerance should outweigh a negative imaging report and prompt a surgical consultation.

To mitigate such diagnostic delays, a systematic clinical approach based on these historical pillars is essential. Suspicion should arise when a patient presents with a long history of severe, paroxysmal digital pain localized to a discrete point. This suspicion is substantially strengthened by eliciting the pathognomonic feature of cold sensitivity, a symptom that should be proactively queried. The physical examination can then provide definitive confirmation by reproducing excruciating pain with gentle pressure from a blunt instrument over the suspected area—the basis of Love's pin test. When these features align, the diagnosis is virtually secured. Complete surgical excision remains the gold standard of treatment, offering immediate and curative pain relief. As recurrence is almost exclusively due to incomplete removal of the lesion, meticulous surgical technique, ideally performed under magnification, is paramount to ensuring a successful outcome [1].

## 7 Conclusion

Glomus tumors, though benign, can cause decades of severe pain if misdiagnosed. This case of a subungual tumor with a 15‐year delay and atypical shoulder radiation highlights that a classic patient history and a meticulous physical examination are paramount and can override inconclusive advanced imaging. Heightened clinical suspicion, guided by an appreciation for both typical and atypical presentations, is essential for timely diagnosis. Complete surgical excision is curative, providing immediate relief and underscoring the importance of early and accurate identification to alleviate profound patient suffering.

## Author Contributions

**Eesha Iqbal:** conceptualization, data curation, formal analysis, investigation, methodology, project administration, resources, software, supervision, validation, visualization, writing – original draft, writing – review and editing. **Muhammad Hamza:** conceptualization, data curation, formal analysis, visualization, writing – original draft, writing – review and editing. **Tariq Rashid:** conceptualization, data curation, formal analysis, resources, software, supervision, visualization, writing – original draft, writing – review and editing. **Laiba Nasir:** conceptualization, data curation, formal analysis, methodology, project administration, visualization, writing – original draft, writing – review and editing. **Fareena Ambreen:** resources, software, supervision, validation, visualization, writing – original draft, writing – review and editing. **Fazeela Bibi:** conceptualization, data curation, resources, software, supervision, validation, visualization, writing – original draft. **Khalil El Abdi:** conceptualization, data curation, supervision, validation, visualization, writing – original draft, writing – review and editing. **Said Hamid Sadat:** writing – original draft, writing – review and editing.

## Funding

The authors have nothing to report.

## Ethics Statement

This case report was conducted in accordance with the ethical principles of the Declaration of Helsinki.

## Consent

Written informed consent was obtained from the patient for publication of this case report and any accompanying images, and all patient data has been anonymized to protect privacy.

## Conflicts of Interest

The authors declare no conflicts of interest.

## Data Availability

The data that support the findings of this study are available from the corresponding author upon reasonable request.
